# Supplementary material for: The Use of Salvage Chemotherapy for Patients with Relapsed Testicular Germ Cell Tumor (GCT) in Canada: A National Survey
Source: Curr Oncol. 2023 Jun 27;30(7):6166–76. doi: 10.3390/curroncol30070458 (PMC10378146; doi:10.3390/curroncol30070458)
Supplement: Supplementary file 1 [file curroncol-30-00458-s001.zip › curroncol-2447443-supplementary.pdf]

The Use of Salvage Chemotherapy for Patients with Relapsed Testicular Germ Cell Tumour in Canada: A National Survey Study Questions:

1. **What is your specialty?**
- Medical Oncology
- Hematology
- Both

2. **Which one of the following best describes your current level of practice?**
- Resident physician
- Fellow physician
- Staff physician

3. **What is the province and cancer center you are currently practicing in?**
- Alberta, Cross Cancer Institute
- Alberta, Jack Ady Cancer Centre
- Alberta, Tom Baker Cancer Centre
- BC Cancer AgencyAbbotsford, Kelowna, Prince George, Surrey, or Victoria
- BC Cancer Agency, Vancouver
- Manitoba, CancerCare Manitoba
- New Brunswick, Dr. Leon-Richard Oncology Center
- New Brunswick, Saint John Regional Hospital
- Newfoundland and Labrador. Dr. H Bliss Murphy Cancer Clinic
- Nova Scotia Cancer CentreQEII or Cape Breton
- Ontario, Grand River Regional Cancer Centre, North East/West Regional Cancer Centre, R.S. McLaughlin Durham Regional Cancer Centre, Royal Victoria Regional Health Centre, Stronach Regional Cancer Centre, Trillium Health Partners, William Osler Health System, or Windsor Regional Cancer Centre
- Ontario, Juravinski Cancer Centre
- Ontario, London Health Sciences Centre
- Ontario, Ottawa Hospital Cancer Centre
- Ontario, Princess Margaret Cancer Center
- PEI Cancer Treatment Centre
- Quebec, Cedars Cancer Center,Centre Hospitalier de Gatineau,Complexe Hospitalier de la Sagamie, Hôpital Charles Lemoyne,Hôpital de Laval,Trois-Rivières, or Rimouski
- Quebec, CHUM – Hopital Notre-Dame
- Quebec, CHUQ - Hôtel-Dieu de Québec
- Quebec, Hôpital Maisonneuve-Rosemont
- Quebec, McGill University Health Centre
- Quebec, Segal Cancer Center / Jewish General Hospital
- Saskatchewan Cancer Agency/ Allan Blair Cancer Centre
- Saskatchewan Cancer Agency/ Saskatoon Cancer Centre
- Other, please specify

4. **How many patients with relapsed germ cell tumors receive salvage chemotherapy at your center (either conventional [CDCT] or high-dose chemotherapy with autologous stem cell transplant [HDCT + ASCT])?**
- <1 case/year

- 1 case/year
- 1-5 cases/year
- 6-10 cases/year

5. Currently, are there any trials available at your center for patients with relapsed germ cell tumor requiring salvage chemotherapy?

- No
- Yes, please describe

6. Which of the following salvage chemotherapy treatments do you oversee at your center?

- Conventional dose chemotherapy (CDCT) only
- High dose chemotherapy (HDCT) + autologous stem cell transplant (ASCT) only
- Both CDCT and HDCT + ASCT

7. At your center, what is the most common conventional dose salvage chemotherapy (CDCT) used for patients with relapsed germ cell tumours?

- TIP (paclitaxel, ifosfamide and cisplatin)
- VIP (etoposide, ifosfamide and cisplatin)
- VeIP (Vinblastine, ifosfamide, and cisplatin)
- PVB (Cisplatin, vinblastine, bleomycin)
- PEI (cisplatin, etoposide, ifosfamide)
- Other regimen, please describe

8. Let's assume you have both salvage chemotherapy options available. How does the International Prognostic Factor Study Group (IPFSG) risk category impact your treatment selection for salvage HDCT + ASCT vs CDCT?(The IPFSG prognostic factors are: primary site, prior response, progression-free interval, AFP and HCG at relapse, presence of liver/bone/brain metastasis, histology; IPFSG prognostic categories: very low, low, intermediate, high, very high risk)

- I am not familiar or aware of the IPFSG risk category.
- Does not impact my treatment selection. I would recommend salvage HDCT + ASCT for all patients.
- Does not impact my treatment selection. I would recommend salvage CDCT for all patients.
- I would recommended salvage CDCT for IPFSG very low and low risk disease, and HDCT + ASCT for all other patients.
- I would recommend salvage CDCT for IPFSG low risk disease only, and HDCT + ASCT for all other patients.
- I use another prognostic risk stratification to select patients for HDCT + ASCT. Please describe.

9. Let's assume you have no resource issues limiting availability of salvage HDCT + ASCT. In your opinion, what is the ideal treatment setting to use salvage HDCT + ASCT for patients with relapsed germ cell tumours?

- First line salvage setting after initial cisplatin-based chemotherapy
- Second line salvage setting after salvage CDCT
- Third line salvage setting or beyond

10. Is salvage HDCT + ASCT available at your center for patients with relapsed germ cell tumor?

- Yes
- No

11. **If salvage HDCT + ASCT is not available at your center, when do you typically refer patients with relapsed germ cell tumour for salvage HDCT + ASCT?**
- Upon first relapse after initial cisplatin-based chemotherapy
- Upon further relapse after salvage CDCT
- I don’t usually refer patients for salvage HDCT + ASCT

12. **How many patients with relapsed germ cell tumors receive salvage HDCT + ASCT at your center?**
- <1 case/year
- 1 case/year
- 1-5 cases/year
- 6-10 cases/year

13. **What percentage of salvage HDCT + ASCT are given at your center in the following treatment settings? (Include a number without decimals 1-100 for each row, all 3 numbers need to add up to 100)**
- First line salvage setting after initial cisplatin-based chemotherapy
- Second line salvage setting after salvage CDCT
- Third line salvage setting or beyond

14. **Are there any clinical scenarios which preclude the use of salvage HDCT + ASCT at your center?**
- Yes. Please describe.
- No

15. **Has the ongoing COVID-19 pandemic influenced the use of salvage chemotherapy at your center?**
- No. Offering both CDCT and HDCT + ASCT the same as before the pandemic.
- Yes. Offering more CDCT and less HDCT + ASCT
- Yes. Offering more HDCT + ASCT and less CDCT
- Other. Please describe

16. **If salvage HDCT + ASCT is planned, is "bridging" conventional dose chemotherapy (CDCT) given at your center while waiting for HDCT to be organized?**
- No. HDCT can be organized within 3 weeks
- No. HDCT takes 3-6 weeks to organize, but no "bridging" CDCT is used
- Yes "bridging" CDCT is used. Please describe the CDCT regimen and number of cycles used.

17. **If “bridging” CDCT is used, which one of the following investigations do you order after completion of “bridging” CDCT, prior to proceeding with salvage HDCT + ASCT?**
- Tumor markers (AFP, bHCG, LDH) only
- Imaging only
- Both tumor markers and imaging

18. **If “bridging” CDCT is used, is disease response (eg. biochemical and/or radiographic) required to proceed with salvage HDCT + ASCT at your center?**
- Always. Patients receive HDCT + ASCT only if evidence of disease response.
- Never. Patients proceed to HDCT + ASCT regardless.
- Case by case discussion. Please describe.

19. **If “bridging” CDCT is used, when is apheresis/collection started following the completion of “bridging” CDCT?**
- Within 4 weeks

- Within 4-6 weeks
- Within 6-8 weeks
- More than 8 weeks
- I don't know

20. **Is neupogen or grastofil used to mobilize peripheral blood stem cells (PBSC) prior to salvage HDCT + ASCT?**
- Yes. Please describe dose and schedule.
- I don't know

21. **What is the minimum number of collected CD34 cells required for salvage HDCT + ASCT to proceed?**
- CD34+ cell count 2 - 3 x 10^6 /kg
- CD34+ cell count 3.1 - 4 x 10^6 /kg
- CD34+ cell count 4.1 - 5 x 10^6 /kg
- CD34+ cell count 5.1 - 6 x 10^6 /kg
- CD34+ cell count > 6 x 10^6 /kg
- I don't know

22. **At your center, when is salvage HDCT and ASCT started after peripheral blood stem cell (PBSC) collection?**
- Within 2 weeks
- Within 2 - 4 weeks
- Within 4 - 6 weeks
- I don't know

23. **At your center, does administration of salvage HDCT + ASCT require planned admission to hospital?**
- Yes
- No

24. **What is the salvage HDCT regimen most commonly used at your center for pateints with relapsed germ cell tumour?**
- Carboplatin Etoposide for 2 cycles
- Carboplatin Etoposide for 3 cycles
- Paclitaxel Ifosfamide for 2 cycles followed by Carboplatin Etoposide for 3 cycles (TI-CE, TIGER trial protocol)
- Other, please describe regimen and number of cycles

25. **Which of the following investigations do you order post first cycle of salvage HDCT?**
- Tumor markers (AFP, bHCG, LDH) only
- Imaging only
- Both tumor markers and imaging
- None

26. **Do the tumor markers and CT results post first cycle of HDCT + ASCT affect your decision to proceed with subsequent cycle of HDCT?**
- Yes. If disease progression, subsequent cycle of HDCT is abandoned.
- No. Patient proceeds with subsequent cycle of HDCT regardless.

- Case by case. Please describe

27. What treatment options do you routinely offer after patients complete salvage HDCT + ASCT?

- Surgical resection for residual disease if feasible
- Maintenance etoposide
- Both above
- None. Surveillance only

28. After completion of salvage HDCT + ASCT, which specialty or specialities follow(s) patients at your center?

- Mainly medical oncology
- Mainly hematology
- Mix of both
- Other, please describe

29. After completion of salvage HDCT + ASCT, how often would you perform the following surveillance investigations within the first year? Please enter a number (1-12) for each.

- Tumor markers every ___ months
- Imaging every ___ months

30. Any other comments about the use of salvage chemotherapy for patients with relapsed germ cell tumours?

- Open-Ended Response
